# MiR-124-3p Suppresses the Dysfunction of High Glucose-Stimulated Endothelial Cells by Targeting G3BP2

**DOI:** 10.3389/fgene.2021.723625

**Published:** 2021-10-08

**Authors:** Haijun Zhao, Yanhui He

**Affiliations:** ^1^Department of Pain, The First Hospital of Jilin University, Changchun, China; ^2^Department of Ophthalmology, The Second Hospital of Jilin University, Changchun, China

**Keywords:** diabetic retinopathy, miR-124-3p, G3BP2, MAPK signaling, high glucose, endothelial cells

## Abstract

**Background:** Diabetic retinopathy (DR) is the most important manifestation of diabetic microangiopathy. MicroRNAs (miRNAs), members of non-coding RNAs, have been frequently reported to regulate various diseases including DR. MiR-124-3p is involved in DR based on bioinformatics. The current study aimed to investigate the role of miR-124-3p in high glucose (HG)-treated human retinal microvascular endothelial cells (HRMECs), an *in vitro* model of DR.

**Methods:** Bioinformatics analysis was applied to reveal the targets downstream miR-124-3p. A series of assays including CCK-8, luciferase reporter, western blot, and tube formation assays were used to explore the function and mechanism of miR-124-3p in HG-stimulated HRMECs.

**Results:** We found out that miR-124-3p was downregulated in HG-stimulated HRMECs. Functionally, miR-124-3p overexpression restrained the HG-induced cell injury of HRMECs. Mechanistically, we predicted 5 potential target mRNAs of miR-124-3p. G3BP stress granule assembly factor 2 (G3BP2) was validated to bind with miR-124-3p. Rescue assays showed that miR-124-3p suppressed cell injury of HG-stimulated HRMECs through G3BP2. In addition, miR-124-3p regulated the p38MAPK signaling pathway by G3BP2, and G3BP2 promoted injury of HG-treated HRMECs through the activation of the p38MAPK signaling pathway.

**Conclusion:** MiR-124-3p suppressed the dysfunctions of HG-treated HRMECs by targeting G3BP2 and activating the p38MAPK signaling. This new discovery provided a potential biomarker for DR treatment.

## Introduction

Diabetic retinopathy (DR) is a common microvascular complication of diabetes (Cheung et al., 2010; Henriques et al., 2015; Diallo et al., 2019). Early features of DR include blood-retina barrier (BRB) breakdown, capillary acellularity and pericyte loss and it has been confirmed that the main factor contributing to the progression of DR is chronic hyperglycemia (Stitt et al., 2016; Powers et al., 2017). In the last decade, significant advances in the diagnosis and treatment of DR have been made (Jenkins et al., 2015; Ebneter and Zinkernagel, 2016). However, DR is still the main cause of vision loss in the world (Ebneter and Zinkernagel, 2016; Horton et al., 2016). Therefore, it is of great significance to deepen our understanding of DR development.

In the early stage of DR, human retinal microvascular endothelial cells (HRMECs), components of the BRB, are impaired by the adverse impact of high glucose (HG), resulting in BRB dysfunction and accelerating DR progression (Strauss, 2005; Miyamoto et al., 2007). In many previous studies, HG-stimulated HRMEC was employed as an *in vitro* model of DR to explore the influences of specific genes on DR (Abu El-Asrar et al., 2016; Gu et al., 2019; Zhu et al., 2019). Similarly, HRMEC was employed as an *in vitro* model of DR in this study.

MicroRNAs (miRNAs) are small and short non-coding RNAs, binding to the 3′-untranslated regions (3′-UTRs) of messenger RNAs (mRNAs) to participate in the regulation of various diseases (Bentwich, 2008; Erson-Bensan, 2014; Mohr and Mott, 2015; Armand-Labit and Pradines, 2017; Lu and Rothenberg, 2018). For example, miR-22 overexpression restrains oxidative stress injury in diabetic cardiomyopathy by targeting Sirt 1 (Tang et al., 2018). MiR-27a-3p attenuates brain injury and blood-brain barrier dysfunction by targeting endothelial AQP11 after intracerebral hemorrhage (Xi et al., 2018). MiR-195-5p facilitates cardiomyocyte hypertrophy via targeting MFN2 and FBXW7 (Wang et al., 2019). Specially, it has been reported that miRNAs are vital players in the regulation of DR (Mastropasqua et al., 2014; Martinez and Peplow, 2019; Satari et al., 2019; Shafabakhsh et al., 2019). Previous studies have demonstrated that miR-124-3p is closely related to several diseases including tuberous sclerosis complex angiomyolipoma and brain injury (Cai et al., 2018; Vuokila et al., 2018; Liang et al., 2019). Importantly, it has been reported that MALAT1 exerts essential effect on DR partly through the regulation of Sag and Guca1a via miR-124-3p (You et al., 2018). Driven by it, we detected the expression of miR-124-3p in HG-stimulated HRMECs and found that HG induced the downregulation of miR-124-3p in HRMECs, which encourages us to further explore its functions in HG-stimulated dysfunctions of HRMECs.

G3BP stress granule assembly factor 2 (G3BP2) has been reported to participate in the regulation of several diseases including cardiac hypertrophy, foot-and-mouth disease, and cancers (Wei et al., 2015; Hong H.Q. et al., 2018; Visser et al., 2019). Particularly, G3BP2 was identified to aggravate the development of diabetic nephropathy (Carney, 2016; Zhao et al., 2016). Based on bioinformatics analysis, G3BP2 is targeted by miR-124-3p. However, no study has been conducted on the role of G3BP2 in DR.

We hypothesized that miR-124-3p can suppress the HG-induced injury in HRMECs and conducted functional assays to confirm it. Moreover, the association between G3BP2 and miR-124-3p was explored. Our research may shed some light on the pathology of DR, which may help develop more effective therapeutic methods to conquer this disease.

## Materials and Methods

### Cells and Cell Culture

The human retinal microvascular endothelial cells (HRMECs; Chinese Academy of Sciences Cell Bank, shanghai, China) were cultured in the Dulbecco’s Modified Eagle Medium (DMEM; Gibco, United States) added with 10% fetal bovine plasma (FBS, Gibco). The HRMECs were incubated in a humidified atmosphere at 37°C containing 5% CO_2_.

### Cell Transfection

The pcDNA3.1/G3BP2 and its negative control (empty pcDNA3.1), miR-124-3p mimics and its negative control (NC mimics) were purchased from GenePharma (Shanghai, China). The above-mentioned vectors were transfected into HRMECs for 24 h using Lipofectamine 3000 (Invitrogen, Carlsbad, CA, United States) according to the manufacturer’s instructions.

### RNA Extraction and Real-Time Quantitative Polymerase Chain Reaction

TRIzol reagent (Invitrogen) was employed to extract total RNA from HRMECs. Next, the extracted RNA was reverse transcribed to cDNA with High Capacity cDNA Reverse Transcription Kits (Applied Biosystems, Foster City, CA, United States). Quantitative PCR was conducted with Power SYBR Green RT-PCR Reagents (Applied Biosystems, Foster City, CA, United States). All reactions were performed on Applied Biosystems 7000 Sequence Detection System (Applied Biosystems, Foster City, CA, United States). Data were processed using the 2^–ΔΔ*Ct*^ method (Livak and Schmittgen, 2001) normalized to U6 or GAPDH. Primers were listed as follows:

miR-124-3p, forward: 5′-TAAGGCACGCGGTGAATG-3′,
reverse: 5′-CAGTGCAGGGTCCGAGGTAT-3′.
U6, forward: 5′-ATACAGAGAAAGTTAGCACGG-3′,
reverse: 5′-GGAATGCTTCAAAGAGTTGTG-3′.
G3BP2, forward: 5′-GTTTGCTGTCTAACAGTGGA-3′,
reverse: 5′-TATTTGGAACAGATCCTTCAGG-3′.
GAPDH, forward: 5′-TCATTTCCTGGTATGACAACGA-3′,
reverse: 5′-GTCTTACTCCTTGGAGGCC-3′.

### Cell Treatment

Human retinal microvascular endothelial cells (HRMECs) in control (Con) group were treated with 5 mM glucose. In mannitol group, cells were treated with 5 mM glucose plus 30 mM mannitol (osmotic control). In high glucose (HG) group, cells were treated with 15 mM, 30 mM, 45 mM glucose. Cells were treated with mannitol and/or glucose for 24 h. HRMECs treated with 30 mM glucose were cultured for 0 h, 12 h, 24 h, 48 h, and 72 h for the detection of cell viability. SB203580 (50 mM; a specific inhibitor of p38 MAPK) was used to treat HRMECs for 60 min as previously described (Hong L. et al., 2018; Chen et al., 2020). SB203580 was bought from Sigma (Shanghai, China) and dissolved in dimethyl sulfoxide (DMSO, Sigma).

### Western Blot

Cell lysate was collected using RIPA lysis buffer. The cell protein mass of each lysate was determined by BCA Protein Assay Reagent (Pierce, IL). Proteins were separated using 10% SDS-polyacrylamide gel electrophoresis (SDS-PAGE), transferred to membranes (0.22 μm, Sigma) and incubated with primary antibodies at 4°C overnight. The primary antibodies are as follows: antibodies against VEGFA (1/1000; ab1316, Abcam), TGFβ1 (1/1000; ab215715, Abcam), Angiopoietin-1 (1/20000; ab183701, Abcam), ZO-1 (1/1000; ab216880, Abcam), Occludin (1/1000; ab216327, Abcam), Claudin-5 (1/1000; ab131259, Abcam), G3BP2 (1/2000; ab86135, Abcam), p-p38 (1/1000; ab195049, Abcam), p38 (1/2000; ab170099, Abcam), p-p53 (1/2000; ab33889, Abcam), p53 (1/1000; ab26, Abcam), and GAPDH (1/500; ab8245, Abcam). Next, the membranes were incubated with HRP-conjugated secondary antibody IgG (1/2000; ab7090, Abcam) at room temperature for 2 h. GAPDH antibody served as a negative control. At last, the protein bands were visualized by an ECL Western Blotting Substrate Kit (ab65623, Abcam) and quantified by the ImageJ software.

### Luciferase Reporter Assay

The 3’UTR of G3BP2 containing miR-124-3p binding site was predicted from the starBase online database. The wild-type (Wt) or the mutant (Mut) 3’UTR of G3BP2 was inserted into pmirGLO vectors (Promega, Madison, MI) to construct the pmirGLO-G3BP2-Wt or Mut vectors. These vectors were then co-transfected with miR-124-3p mimics or NC mimics into HRMECs by Lipofectamine 3000 (Invitrogen). The luciferase activities were detected 48 h after transfection with the Dual-Luciferase Reporter Assay System (Promega, Madison, WI, United States). The firefly luciferase activity was normalized to *Renilla* luciferase activity.

### CCK-8 Assay

Cell viability was detected using a Counting Kit-8 (CCK-8) (Dojindo, Tokyo, Japan). HRMECs (1 × 10^3^ cells/well) stimulated by HG for 0 h, 12 h, 24 h, 48 h, 72 h were plated into a 96-well plate. Next, each well was supplemented with CCK-8 solution (10 μL) for 4 h of incubation at 37°C, and the absorbance values were detected at 450 nm.

### Tube Formation Assay

The tube formation ability was determined by performing tube formation assay. Growth Factor Reduced Matrigel matrix (Corning) (300 μL) was put on the bottom of a 24-well plate, and HRMECs (2 × 10^4^ cells/per well) were seeded into wells. After 20 h, capillary-like structures were visualized with a Nikon Eclipse Ti inverted microscope (Nikon). For each well, at least six different fields were randomly chosen for observation. Finally, meshes and branch length of the capillary-like structures were evaluated employing ImageJ software (version1.49p; NIH, Bethesda, MD, United States).

### Statistical Analysis

All experiments were performed three times. Statistical analysis was conducted by SPSS 13.0. The data were shown as the mean ± SD. Differences between two groups were analyzed employing the two-tailed unpaired *t*-test. Multiple comparisons were calculated using one-way analysis of variance (ANOVA). *P* < 0.05 was considered statistically significant.

## Results

### MiR-124-3p Was Downregulated in HG-Stimulated HRMECs

**FIGURE 1:**
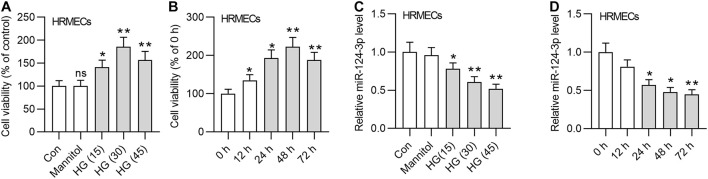
MiR-124-3p was downregulated in HG-stimulated HRMECs. **(A)** The viability of HRMECs treated with 5 mM glucose, 5 mM glucose plus 30 mM mannitol, 15 mM glucose, 30 mM glucose, 45 mM glucose for 24 h was detected by CCK-8 assay. **(B)** The viability of HRMECs treated with 30 mM glucose for 0 h, 12 h, 24 h, 48 h, and 72 h was detected by CCK-8 assay. **(C)** The expression level of miR-124-3p in HRMECs treated with 5 mM glucose, 5 mM glucose plus 30 mM mannitol, 15 mM glucose, 30 mM glucose, 45 mM glucose for 48 h was detected with RT-qPCR analysis. **(D)** The expression level of miR-124-3p in HRMECs treated with 30 mM glucose for 0 h, 12 h, 24 h, 48 h, and 72 h was detected with RT-qPCR analysis. ns indicates no significance, *P < 0.05, **P < 0.01, ***P < 0.001.

First, we detected the viability of HRMECs in the medium containing glucose of different concentrations (15 mM, 30 mM, and 45 mM). The results demonstrated that the viability of HRMECs was enhanced by HG treatment for 24 h, and 30 mM HG achieved the best effects (Figure 1A). We employed 30 mM HG to treat HRMECs in the following experiments. Next, we observed that the viability of HRMECs was significantly enhanced by the increasing time of HG (30 mM) treatment and the viability reached the highest at 48 h (Figure 1B), so we treated cells with HG for 48 h in the following assays. In addition, we detected the expression of miR-124-3p by increased concentrations of glucose, and found that miR-124-3p level in HRMECs was gradually decreased by increased concentrations of glucose (Figure 1C). Moreover, miR-124-3p level in HRMECs was also time-dependently downregulated by HG stimulation (Figure 1D). The present study is based on the bioinformatics-based results of a previous study (You et al., 2018), which showed that miR-155-5p, miR-1-3p, miR-122-5p, miR-223-3p, miR-125b-5p, and miR-124-3p are potential links between MALAT1 and five visual perception-related genes (PDE6G, GUCA1A, RHO, SAG, and PRPH2) in DR. Expression of abovementioned molecules in the *in vitro* model of DR needs validation. We found that miR-155-5p, miR-122-5p, miR-223-3p, miR-125b-5p were all downregulated in HG-treated HRMECs, while expression of miR-1-3p showed no significant difference between control and HG groups (Supplementary Figure 1A). Expression of MALAT1, PDE6G, GUCA1A, RHO, SAG, and PRPH2 in HRMECs was increased by HG stimulation (Supplementary Figure 1B), which was consistent with the previous study.

### MiR-124-3p Overexpression Restrained the HG-Induced Cell Injury of HRMECs

**FIGURE 2:**
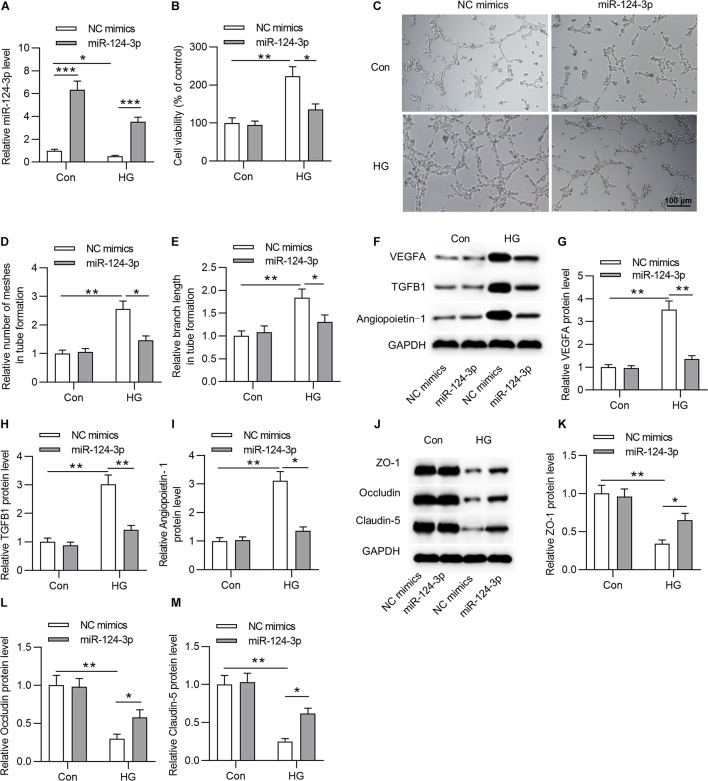
MiR-124-3p overexpression restrained HG-induced cell injury of HRMECs. **(A)** The overexpression efficiency of miR-124-3p mediated by transfection of miR-124-3p mimics for 24 h in HRMECs was assessed by RT-qPCR analysis. **(B)** The effect of miR-124-3p overexpression on the viability of HRMECs in the control group (5 mM glucose) and HG group (30 mM glucose) was evaluated by CCK-8 assay. **(C–E)** The mesh number and branch length of HRMECs upon the introduction of HG (30 mM glucose) for 48 h was detected by the tube formation assay. **(F–I)** Western blot analysis measured the levels of proteins associated with angiogenesis (VEGFA, TGFB1 and Angiopoietin-1) in NG (5 mM glucose) or HG (30 mM glucose)-stimulated HRMECs followed by the transfection of miR-124-3p mimics for 24 h. **(J–M)** The levels of proteins associated with blood-ocular barrier (ZO-1, Occludin and Claudin-5) in NG (5 mM glucose) or HG (30 mM glucose)-stimulated HRMECs followed by the transfection of miR-124-3p mimics for 24 h were analyzed by western blot analysis. *P < 0.05, **P < 0.01, ***P < 0.001.

To explore the biological function of miR-124-3p in HRMECs, we first overexpressed miR-124-3p by transfecting miR-124-3p mimics into NG or HG treated HRMECs (Figure 2A). Subsequently, the CCK-8 assay showed that, compared with control group, overexpressed miR-124-3p reduced the viability of HG-stimulated HRMECs (Figure 2B). Enhanced expression of miR-124-3p decreased the number of meshes and branch length in tube formation upon the introduction of HG into HRMECs (Figures 2C–E). Similarly, the levels of proteins associated with angiogenesis (VEGFA, TGFB1 and Angiopoietin-1) were declined in response to miR-124-3p mimics in HG-stimulated HRMECs (Figures 2F–I). Moreover, western blot analysis revealed that miR-124-3p mimics enhanced the level of proteins associated with blood-ocular barrier (ZO-1, Occludin and Claudin-5) in HG-stimulated HRMECs (Figures 2J–M). To sum up, miR-124-3p overexpression restrained cell injury of HG-stimulated HRMECs.

### MiR-124-3p Interacted With G3BP2 and Degraded It

**FIGURE 3:**
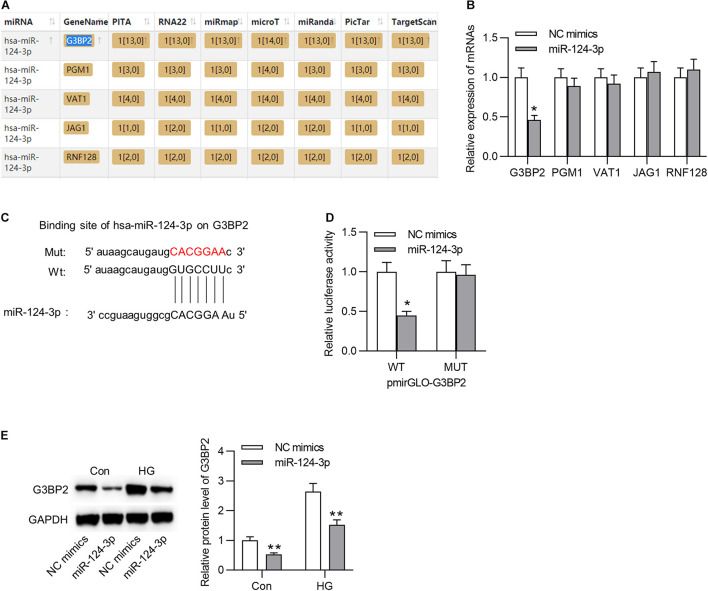
MiR-124-3p interacted with G3BP2 to promote its degradation. **(A)** The mRNAs possessing binding site with miR-124-3p were predicted from starBase. **(B)** The effect of transfection of miR-124-3p mimics for 24 h on the expressions of the 5 candidate mRNAs in HG (30 mM glucose)-stimulated HRMECs was detected with RT-qPCR analysis. **(C)** The binding site between miR-124-3p and G3BP2 3′UTR was predicted from starBase. **(D)** The HRMECs were co-transfected with miR-124-3p mimics (or NC mimics) and pmirGLO-G3BP2-Wt (or pmirGLO-G3BP2-Mut) vectors for 48 h, and then relative luciferase activities were detected. **(E)** The effect of transfection of miR-124-3p mimics for 24 h on the protein level of G3BP2 in NG (5 mM glucose) or HG (30 mM glucose)-stimulated HRMECs was detected by western blot analysis. *P < 0.05, **P < 0.01.

Subsequently, we aimed to probe into the underlying molecular regulatory mechanism of miR-124-3p in HG-stimulated HRMECs. First, we employed starBase online tool^1^ to search the mRNAs that shared binding sites with miR-124-3p (condition: overlapped mRNAs from databases of PITA, RNA22, miRmap, microT, miRanda, PicTar, and TargetScan), and 5 mRNAs were identified (Figure 3A). Next, the expression levels of the 5 candidates were detected in HG-stimulated HRMECs after the transfection of miR-124-3p mimics. Results suggested that miR-124-3p mimics caused an obvious decline of G3BP2 expression (Figure 3B). The binding site between miR-124-3p and G3BP2 was exhibited in Figure 3C. Luciferase reporter assay demonstrated that miR-124-3p mimics resulted in a distinct decrease of the activity of G3BP2-WT and exerted no significant effects in G3BP2-MUT (Figure 3D), implying the binding of miR-124-3p with G3BP2 3′UTR. Furthermore, we noted that miR-124-3p expression reduced the protein level of G3BP2 in HG-stimulated HRMECs (Figure 3E). Overall, miR-124-3p targeted G3BP2 in HG-stimulated HRMECs.

### MiR-124-3p Suppressed Cell Injury Through G3BP2 in HG-Stimulated HRMECs

**FIGURE 4:**
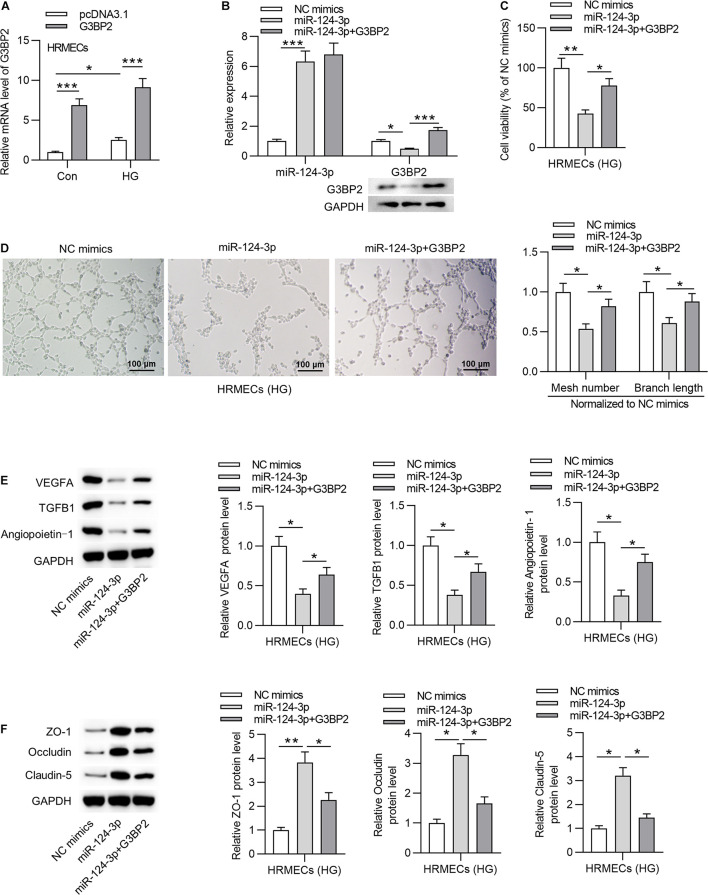
MiR-124-3p suppressed cell injury through G3BP2 in HG-stimulated HRMECs. **(A)** HRMECs were stimulated with NG (5 mM glucose) or HG (30 mM glucose) for 48 h and transfected with pcDNA3.1-G3BP2 for 24 h. The overexpression efficiency of G3BP2 was assessed by RT-qPCR analysis. **(B)** Expression of miR-124-3p in HRMECs after transfection of miR-124-3p mimics or co-transfection of miR-124-3p + G3BP2 for 24 h was evaluated by RT-qPCR analysis, while that of G3BP2 was evaluated by RT-qPCR and western blotting. **(C)** The viability of HG (30 mM glucose)-stimulated HRMECs for 48 h followed by the transfection of miR-124-3p mimics or co-transfection of miR-124-3p + G3BP2 for 24 h was evaluated by CCK-8 assay. **(D)** The mesh number and branch length of HG (30 mM glucose)-stimulated HRMECs for 48 h after transfection of indicated plasmids for 24 h was revealed by tube formation assay. **(E)** Western blot analysis measured the levels of proteins associated with angiogenesis (VEGFA, TGFB1 and Angiopoietin-1) in HG (30 mM glucose)-stimulated HRMECs for 48 h followed by transfection of miR-124-3p mimics or co-transfection of miR-124-3p + G3BP2 for 24 h. **(F)** The levels of proteins associated with blood-ocular barrier (ZO-1, Occludin and Claudin-5) in HG (30 mM glucose)-stimulated HRMECs for 48 h followed by transfection of miR-124-3p mimics or co-transfection of miR-124-3p + G3BP2 for 24 h were analyzed by western blot analysis. *P < 0.05, **P < 0.01, ***P < 0.001.

To investigate whether miR-124-3p regulates cell injury through G3BP2 in HG-stimulated HRMECs, we performed rescue assays. First, we effectively overexpressed G3BP2 by the transfection of pcDNA3.1/G3BP2 into HG-stimulated HRMECs (Figure 4A). Transfection of pcDNA3.1/G3BP2 had no significant effects on miR-124-3p expression and rescued the miR-124-3p-mediated degradation on G3BP2 mRNA and protein (Figure 4B). Next, we observed that G3BP2 overexpression offset the inhibitive effect of miR-124-3p overexpression on cell viability of HG-stimulated HRMECs (Figure 4C). Enhanced expression of G3BP2 partially recovered the miR-124-3p overexpression-induced decrease of mesh number and branch length in tube formation assay (Figure 4D). Meanwhile, miR-124-3p overexpression-induced decrease in the levels of proteins associated with angiogenesis (VEGFA, TGFB1 and Angiopoietin-1) was partially rescued by G3BP2 overexpression in HG-stimulated HRMECs (Figure 4E). The increase in the levels of proteins associated with blood-ocular barrier (ZO-1, Occludin and Claudin-5) caused by upregulated miR-124-3p was partially restored by G3BP2 overexpression in HG-stimulated HRMECs (Figure 4F). In summary, overexpressed G3BP2 counteracted the effect of miR-124-3p overexpression on the dysfunctions of HG-stimulated HRMECs.

### MiR-124-3p Inhibited the p38MAPK Signaling Pathway Through G3BP2

**FIGURE 5:**
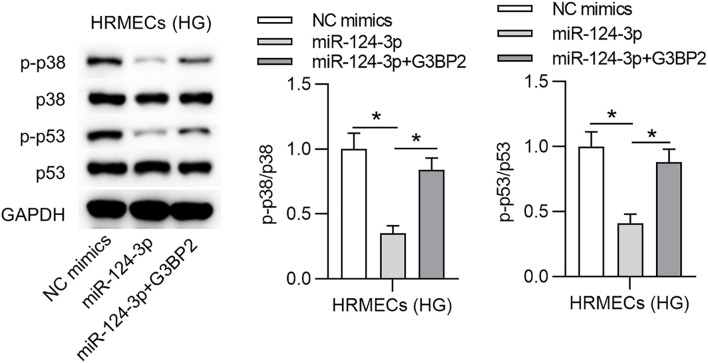
MiR-124-3p inhibited the p38MAPK signaling pathway through G3BP2. The levels of proteins of p38, p53, phosphorylated p53 and p38 in HG (30 mM glucose)-stimulated HRMECs for 48 h followed by transfection of miR-124-3p mimics or co-transfection of miR-124-3p + G3BP2 for 24 h were analyzed by western blot analysis. *P < 0.05.

A previous study has demonstrated that G3BP2 is involved in the regulation of the p38MAPK signaling pathway, and p53 is a downstream target of p38 mitogen-activated protein kinase (p38MAPK) signaling (Zhao et al., 2016). Accordingly, we hypothesized that miR-124-3p regulated p38MAPK signaling pathway through G3BP2 in HG-stimulated HRMECs. With western blot analysis, we observed that miR-124-3p overexpression reduced the protein levels of phosphorylated p53 and p38, which were then partially recovered by overexpressed G3BP2, suggesting that miR-124-3p regulated the p38MAPK signaling pathway through G3BP2 (Figure 5).

### G3BP2 Promoted Cell Injury of HG-Treated HRMECs Through the p38MAPK Signaling Pathway

**FIGURE 6:**
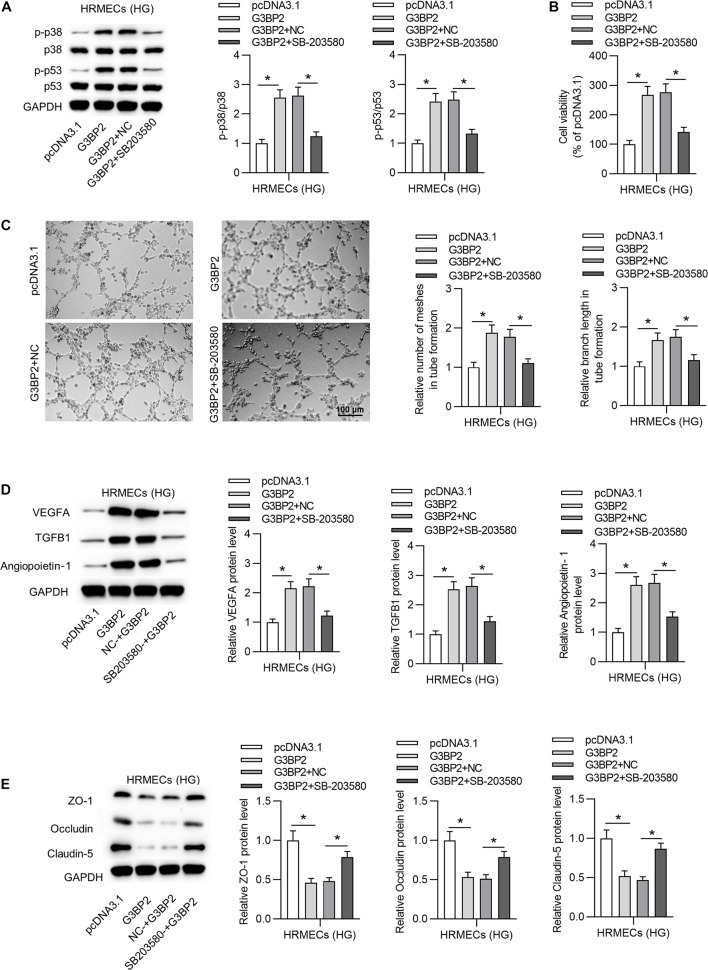
MiR-124-3p promoted HG-induced injury of HRMECs through the p38MAPK signaling pathway. **(A)** The protein levels of p38, p53, phosphorylated p53 and p38 in HG (30 mM glucose)-stimulated HRMECs for 48 h followed by the transfection of pcDNA3.1/G3BP2, or cotreatment of pcDNA3.1/G3BP2 + SB203580 were analyzed by western blot analysis. **(B)** The viability of HG (30 mM glucose)-stimulated HRMECs followed by different treatments was evaluated by CCK-8 assay. **(C)** The mesh number and branch length of HG (30 mM glucose)-stimulated HRMECs after different treatments were detected by tube formation assay. **(D)** Western blot analysis measured the levels of proteins associated with angiogenesis (VEGFA, TGFB1 and Angiopoietin-1) in HG (30 mM glucose)-stimulated HRMECs in the four groups. **(E)** The levels of proteins associated with blood-ocular barrier (ZO-1, Occludin and Claudin-5) in HG (30 mM glucose)-stimulated HRMECs in different groups were analyzed by western blot analysis. *P < 0.05.

To investigate whether G3BP2 regulates cell injury through the p38MAPK signaling pathway in HG-stimulated HRMECs, we performed rescue assays. First, we found that G3BP2 overexpression-induced increase of the protein levels of phosphorylated p53 and p38 was counteracted by the introduction of SB203580 in HG-stimulated HRMECs (Figure 6A). Next, we observed that the introduction of SB203580 offset the promotive effect of G3BP2 overexpression on the viability of HG-stimulated HRMECs (Figure 6B). G3BP2 overexpression-induced elevation of mesh number and branch length in tube formation of HG-stimulated HRMECs was offset by SB203580 (Figure 6C). Meanwhile, G3BP2 overexpression-induced increase in the levels of proteins associated with angiogenesis (VEGFA, TGFB1 and Angiopoietin-1) was rescued by the treatment of SB203580 in HG-stimulated HRMECs (Figure 6D). The decrease in the levels of proteins associated with blood-ocular barrier (ZO-1, Occludin and Claudin-5) caused by upregulated G3BP2 was partially restored by SB203580 in HG-stimulated HRMECs (Figure 6E). In summary, G3BP2 contributed to HG-stimulated injury of HRMECs through the activation of the p38MAPK signaling pathway.

## Discussion

Since HRMECs are considered as the major targets of hyperglycemic injury (Chen et al., 2017), the exploration of the role of miRNAs that regulate HRMECs is of great importance for the understanding of DR. The current study aimed to explore the role of miR-124-3p in HG-stimulated HRMECs, an *in vitro* model of DR. Our data exhibited the vital role of miR-124-3p in suppressing the dysfunctions of HG-stimulated HRMECs.

First, our findings demonstrated that miR-124-3p was downregulated in HG-stimulated HRMECs. MiRNAs are major regulators in a variety of diseases (Kaul and Krams, 2015; Trionfini and Benigni, 2017). Specially, it has been confirmed that miRNAs exert pivotal functions in the initiation and development of DR. For example, miR-138-5p exerts a protective role the early DR by regulating NOVA1 (Bao and Cao, 2019). Serum miR-122 levels is associated with DR (Pastukh et al., 2019). MiR-1273g-3p is involved in DR development (Ye et al., 2017). Recently, it has been reported that miR-124-3p participates in the regulation of various diseases, such as traumatic brain injury (Schindler et al., 2020) and neuropathic pain (Zhang et al., 2019). Importantly, miR-124-3p was reported to be associated with DR (You et al., 2018).

Subsequently, experimental data revealed that miR-124-3p overexpression restrained the HG-induced cell injury of HRMECs by suppressing tube formation ability, reducing pro-angiogenic factors and increasing tight junction proteins. Consistent with this study, previous research has shown that miR-124-3p restrains cell injury in some other diseases, for example, miR-124-3p alleviates the neuronal injury of SH-SY5Y cells induced by MPP^+^ (Geng et al., 2017). MiR-124-3p inhibits cell injury in traumatic brain injury (Vuokila et al., 2018; Schindler et al., 2020). MiR-124-3p alleviates neuronal apoptosis induced by mechanical injury (Su et al., 2019).

Additionally, in this study, G3BP2 was identified as a downstream target of miR-124-3p in HRMECs. G3BP2 is found to be involved in diabetic nephropathy (Carney, 2016; Zhao et al., 2016). We identified that G3BP2 is upregulated in HG-stimulated HRMECs, which indicated the potential involvement of G3BP2 in DR. G3BP2 overexpression rescued the suppressive effects of miR-124-3p on HG-induced injury of HRMECs by promoting tube formation ability, increasing pro-angiogenic factors and decreasing tight junction proteins. Moreover, the p38MAPK signaling pathway is frequently reported to be activated during DR (Zhang et al., 2013; Dong et al., 2020; Liu et al., 2020). A previous research has reported that G3BP2 is involved in the regulation of the p38MAPK signaling pathway (Zhao et al., 2016). In this study, we observed that G3BP2 promoted the HG-induced injury of HRMECs by the p38MAPK pathway. MiR-124-3p suppressed the ratio of p-p38/p38 and p-p53/p53, and the trend was rescued by G3BP2, indicating that miR-124-3p suppressed the p38MAPK signaling pathway by G3BP2.

## Conclusion

The current study provided an evidence that miR-124-3p suppressed the dysfunction of HG-stimulated HRMECs by targeting G3BP2 and suppressing MAPK signaling pathway. Our finding pointed out the potential of miR-124-3p in the development of novel therapeutic methods for DR.

## Data Availability Statement

The raw data supporting the conclusions of this article will be made available by the authors, without undue reservation.

## Author Contributions

HZ and YH wrote first draft of the manuscript and commented on previous versions of the manuscript. Both authors read and approved the final manuscript, contributed to the study conception and design, material preparation, and data collection and analysis.

## Conflict of Interest

The authors declare that the research was conducted in the absence of any commercial or financial relationships that could be construed as a potential conflict of interest.

## Publisher’s Note

All claims expressed in this article are solely those of the authors and do not necessarily represent those of their affiliated organizations, or those of the publisher, the editors and the reviewers. Any product that may be evaluated in this article, or claim that may be made by its manufacturer, is not guaranteed or endorsed by the publisher.

## Footnotes

1
http://starbase.sysu.edu.cn

## Supplementary Material

Supplementary Figure 1 **(A)** Relative expression of miR-155-5p, miR-1-3p, miR-122-5p, miR-223-3p, miR-125b-5p in HRMECs after stimulation of HG (30 mM glucose) for 48 h was measured by RT-qPCR analysis. **(B)** Relative expression of MALAT1, PDE6G, GUCA1A, RHO, SAG, PRPH2 in HRMECs after stimulation of HG (30 mM glucose) for 48 h was measured by RT-qPCR analysis. **P* < 0.05, ***P* < 0.01, ****P* < 0.001.
