# Supplementary figures and images for: MiR-124-3p Suppresses the Dysfunction of High Glucose-Stimulated Endothelial Cells by Targeting G3BP2
Source: Front Genet. 2021 Oct 8;12:723625. doi: 10.3389/fgene.2021.723625 (PMC8531496; doi:10.3389/fgene.2021.723625)

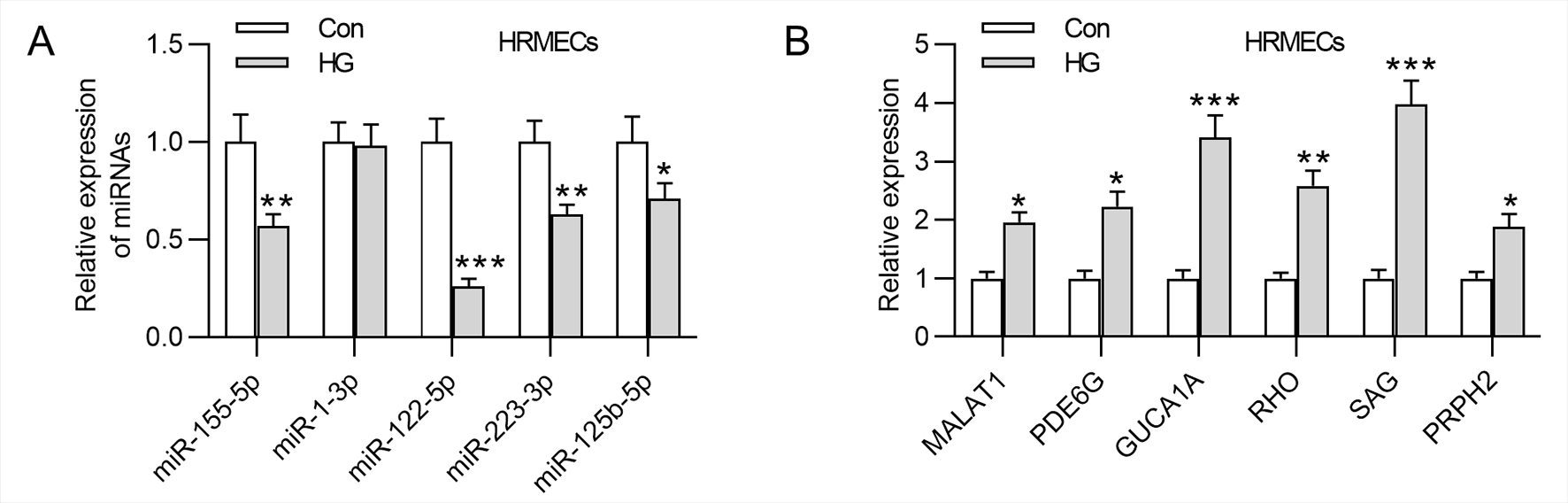

Supplement: Supplementary Figure 1 — (A) Relative expression of miR-155-5p, miR-1-3p, miR-122-5p, miR-223-3p, miR-125b-5p in HRMECs after stimulation of HG (30 mM glucose) for 48 h was measured by RT-qPCR analysis. (B) Relative expression of MALAT1, PDE6G, GUCA1A, RHO, SAG, PRPH2 in HRMECs after stimulation of HG (30 mM glucose) for 48 h was measured by RT-qPCR analysis. *P < 0.05, **P < 0.01, ***P < 0.001. [file Image_1.TIF]

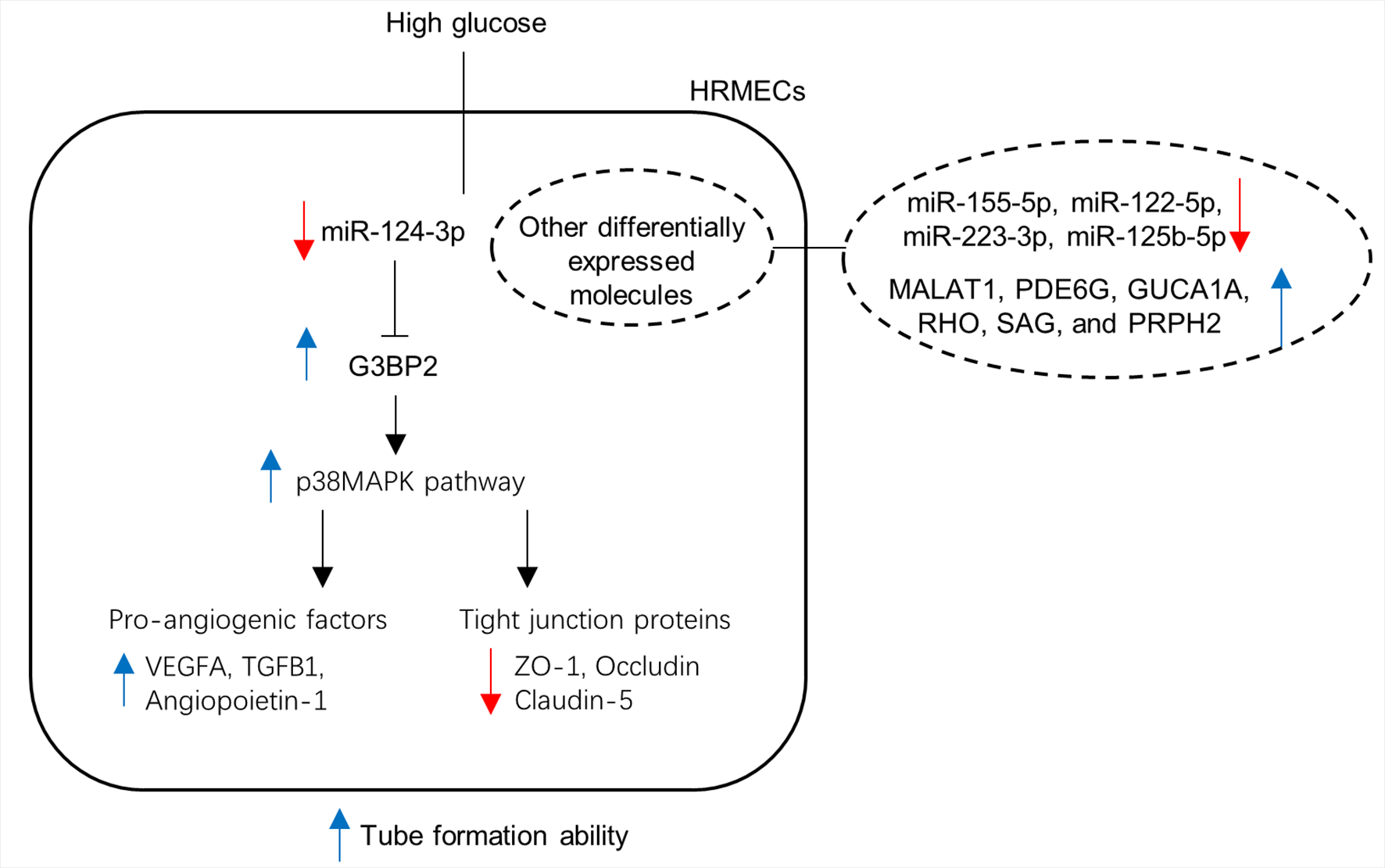

Supplement: Supplementary file 2 [file Image_2.TIF]
